# Modulation of insulin/IGFs pathways by sirtuin-7 inhibition in drug-induced chemoreistance

**DOI:** 10.1186/1746-1596-9-94

**Published:** 2014-05-22

**Authors:** Ahmad Aljada, Ayman M Saleh, Salem Al Suwaidan

**Affiliations:** 1 Department of Basic Medical Sciences, College of Medicine, King Saud bin Abdulaziz University for Health Sciences, P. O. Box 22490, Riyadh 11426, Kingdom of Saudi Arabia; 2 King Abdullah International Medical Research Center (KAIMRC), King Abdulaziz Medical City, National Guard Health Affairs, P. O. Box 22490, Riyadh 11426, Kingdom of Saudi Arabia

**Keywords:** Drug-induced resistance, Stress induced premature senescence, Sirtuin-7, Insulin/IGF

## Abstract

**Background:**

Insulin and insulin-like growth factors (IGFs) are key regulators of metabolism and growth. Recent evidences suggest a key role of these pathways in non-classical tissues and the metabolic pathways by which these hormones exert their effects in neoplasia is unclear.

**Aims:**

To study insulin/IGFs pathways in drug sensitive and resistant cancer cells representing breast cancer (MCF-7), osteosarcoma (SaOS-2), and ovarian cancer (A2780) and to examine the effect of Sirtuin-7 (Sirt7) inhibition on insulin/IGFs pathways in MCF-7 cell line.

**Methods:**

Drug resistant cells were generated by continuous incubation of parental cell lines with stepwise increases in Doxorubicin or Cisplatin over a period of 3 to 6 months. MCF-7 cells were transfected with cloned hairpin siRNA template for Sirt7 using the Amaxa GmbH transfection system. mRNA expression of Sirt7, INSR, IRS-1, IRS-2, IRS-4, IGF-1, IGF-2, MDR-1, MRP-1, BCRP was measured by qPCR and Sirt7 by standard Western blotting. FITC-insulin uptake was imaged with Leica Confocal Microscope.

**Results:**

Insulin receptor (INSR), insulin receptor substrate-1 (IRS-1) were inhibited in drug-induced resistance, whereas IRS-2 was significantly induced in all the chemoresistant cells tested when compared to their parental counterparts. IGF-1 and IGF-2 were also upregulated in all the drug resistant cells tested. Sirt7 was significantly reduced in all chemoresistant cells tested. Knockdown of Sirt7 expression in human breast MCF-7 cell line by siRNA induced premature senescence-like phenotype and multi-drug resistance, suggesting that this gene may play an active role in regulating cancer cell response to stress. Suppression of Sirt7 selectively inhibited INSR and IRS-1, whereas it had minimal effect on that of IRS-2. Sirt7 suppression in MCF-7 also inhibited insulin uptake. Additionally, Sirt7 inhibition upregulated IGF-1, IGF-2 and IGFR expression.

**Conclusion:**

Our data demonstrate that stress-induced Sirt7 inhibition significantly increases stress resistance and modulates insulin/IGF-1 signaling pathways. More importantly, this study links Sir2 family proteins to insulin/IGF signaling in drug-induced stress resistance in neoplasia.

**Virtual Slides:**

The virtual slide(s) for this article can be found here: http://www.diagnosticpathology.diagnomx.eu/vs/1135426681234493

## Background

The autocrine and paracrine secretion of insulin and insulin-like growth-factors (IGFs) 1 and 2 are key regulators of metabolism and growth, which subserve energy production and growth stimulation, respectively, in cancer cells. Several recent evidences suggest a key role of these pathways in non-classical tissues. It is unclear if these hormones exert their effects in a similar manner in neoplasia. Receptors for IGFs and insulin are widely expressed on normal and cancerous tissues. The insulin receptor (INSR) and the IGF-1R are both tyrosine kinase receptors and are structurally similar and activate almost identical intracellular signaling events. Insulin and IGFs receptors have been reported in various cancers including breast [1-9], lung [10], colon [11], melanoma [12], cervix [12], renal cell carcinoma [13], fibrosarcoma [14], Hodgkin’s lymphoma [15], insulinoma [16], as well as in one hematologic malignancy, lymphoblastic leukemia [14]. INSR and IGFRs have likewise been well characterized on the cell membranes of these cancers [11,17-19]. The classical view presents the INSR as being responsible for the metabolic functions and IGF-1R being responsible for the growth, proliferation, protection against apoptosis. These differences may be explained partially by the slight structural differences and tissue distribution; however, a rational explanation for the divergent biological effects is the interactions with specific substrates. Although there are two receptors for IGFs, IGF-1R and IGF-2R, IGF-2R does not transduce a signal and acts to reduce the bioavailability of IGF-2 by sequestering it away from IGF-1R and thus acts as a tumor suppressor [20]. INSR, on the other hand, has two isoforms. INSR-A isoform is commonly expressed by neoplastic tissue whereas INSR-B isoform is commonly expressed by classic-insulin sensitive tissue [21,22]. This preferential expression of INSR isoforms is unclear. Insulin is expressed exclusively by pancreatic β cells whereas IGF-1 and IGF-2 are produced in the liver and neoplastic tissue. *IGF-2* gene is imprinted and its overexpression in neoplasia could result from the loss of imprinting. Additionally, the activity of IGFs is modulated by IGFBPs which limit IGF access to IGF-1R and thus inhibiting IGFs. However, overexpression of certain IGFBPs, in particular IGFBP2 and IGFBP5, results in increased activity of IGF [23,24].

The role of Sirt7 in cancer, an aging-associated disease, is still poorly understood. Sirt7 is associated with active rRNA genes (rDNA) and histones [25]. Overexpression of Sirt7 increased RNA polymerase I (Pol I)-mediated transcription, whereas knockdown of Sirt7 or inhibition of its catalytic activity resulted in decreased association of pol I with rDNA and reduced pol I transcription. Depletion of Sirt7 stopped cell proliferation and triggered apoptosis [25]. We have recently demonstrated that Sirt7 is inhibited in drug-induced resistance. Inhibition of Sirt7 induces stress-induced premature senescence (SIPS) leading to aggressive tumor behavior [26].

In this study, we investigated insulin/IGF pathway in drug-induced resistance and the relationship of Sirt7 to this pathway since it has been shown that reduction in insulin/IGF-1 signaling extends the lifespan of *C. elegans*, *Drosophila* and mice [27-31]. Several studies have also shown that reduced insulin/IGF-1 signaling protects against oxidative damage and other forms of stress [27,28,30]. On the other hand, increased levels of IGF-1 are associated with malignant and non-malignant tumorogenesis [32-34]; and IGF-1 has been shown to inhibit chemotherapy-induced apoptosis by activation of the phosphatidylinositol 3-kinase (PI3K)-Akt pathway. Additionally, inhibition of IGF-1 receptor has been shown to increase the efficacy of etoposide and carbaplatin [35,36]. We hypothesized that stress-induced inhibition of Sirt7, results in modulation of insulin/IGF pathways suggesting a link between these pathways.

## Methods

### Establishment of chemoresistant cells

Human breast cancer MCF-7 and osteosarcoma SaOS-2 cell lines were purchased from the ATCC. The ovarian cancer A2780 cells and their corresponding Cisplatin resistant cells were a generous gift from Dr. Mary J Hendrix (Children’s Memorial Hospital, Chicago, IL). Doxorubicin-resistant MCF-7 and SaOS-2 cells were generated by continuous incubation of parental cell lines with stepwise increases in doxorubicin concentration over a period of 3 to 6 months. Cells were grown in Minimum Essential Medium (MEM, Eagle) with 2 mM L-glutamine and Earle’s BSS adjusted to 1.5 g/L sodium bicarbonate, 0.1 mM non-essential amino acids, 1.0 mM sodium pyruvate, and 10% fetal bovine serum.

### Cell transfection

MCF-7 cells were transfected with pSilencer 4.1-CMV neo vector (Ambion, Inc., Austin, TX) containing the cloned hairpin siRNA template for Sirt7 using the Amaxa GmbH transfection system (Amaxa, Inc., Gaithersburg, MD). Three constructs were generated targeting three different regions of Sirt7. Mock cells were transfected with pSilencer 4.1-CMV neo-vector that expresses a hairpin siRNA with limited homology to any known sequences in the human. This method exhibited 80% transfection efficiency in MCF-7.

### mRNA Quantification by Real-Time RT-PCR

Total RNA was isolated using the Ambion Aqueous kit (Ambion). The quality and quantity of the isolated RNA was determined using a Bio-Rad Experion automated electrophoresis system (Hercules, CA). Then, 1 μg of total RNA was reverse-transcribed using Advantage RT-for-PCR Kit (Clontech; Mountain View, CA). Real time RT-PCR was performed with a Cepheid Smart Cycler (Sunnyvale, CA), using 2 μL cDNA, 10 μL Sybergreen Master mix (Qiagen; Valencia, CA) and 0.5 μL of 20 μM gene-specific primers: Sirt7-F: CCTCCTGCGTTCCCAACAG; Sirt7-R: GCTTCCCAGTTCAGAGGCT; INSR-F: CGTCCCCAGAAAAACCTCTTC; INSR-R: ACGGCCACCGTCACATTC; IRS-1-F: CGCCGCTCAAGTGAGGATTTAAGC; IRS-1-R: ATGCATCGTACCATCTACTGATGAGG; IRS-2-F: ACAATGGTGACTACACCGAG; IRS-2-R: CTGCTTTTCCTGAGAGAGAC; IRS-4-F: CTTCACTCGCGACCAAGCGACAAG; IRS-4-R: GTGCCCATGCTTCTGTTTCCGCAG; IGF-1-F: GATCCTTTGCTCTGCACGAGTTACCTG; IGF-1-R: TTTGTGGCTCTTGAGAGGCAGGGACT; IGF-2-F: CCTCCAGTTCGTCTGTGGG; IGF-2-R: CACGTCCCTCTCGGACTTG; MDR-1-F (ABCB1): TGACATTTATTCAAAGTTAAAAGCA; MDR-1-R: TAGACACTTTATGCAAACATTTCAA; MRP-1-F (ABCC1): CGGAAACCATCCACGACCCTAA; MRP-1-R: TCATGAGGAAGTAGGGCCCAAA; BCRP-F (ABCG2): CCGCGACAGTTTCCAATGACCT; BCRP-R: GCCGAAGAGCTGCTGAGAACTGTA; GAPDH-F: TGCACCACCAACTGCTTAGC; GAPDH-R: GGCATGGACTGTGGTCATGAG; β-actin-F: TGACTGACTACCTCATGAAGATCC; β-actin-R: CCATCTCTTGCTCGAAGTCCAG; The specificity and size of the PCR products were tested by adding a melt curve at the end of the amplifications, analysis on a 2% agarose gel and sequencing of the bands. All values were normalized to GAPDH and β-actin.

### Sirt7, β-actin and GAPDH Western blotting

Western blotting was carried out by standard Western blotting technique. Total cellular homogenates were prepared using Pierce mammalian extraction reagent (Pierce, Rockford, IL), according to the instructions provided by the manufacturer. Protein quantization was performed by BCA method (Pierce Biotechnology, Inc., Rockford, IL). A total of 60 μg of cell lysates were boiled in 2X SDS buffer (100 mM Tris–HCl, 4% SDS, 20% glycerol, 0.06% bromophenol blue, and 200 mM DTT), proteins separated by SDS-PAGE and then transferred to PVDF membrane. Membranes were blocked in blocking buffer (5% dried milk powder, 1%TBS-T) after which the primary antibodies against GAPDH or β-actin (Santa Cruz Biotechnology Inc., Santa Cruz, CA) or Sirt7 (Sigma, St. Louis, MO) were added. The washed blots were then incubated in secondary antibody (goat anti-rabbit; Bio-Rad) diluted to 1:10000 and proteins were visualized using SuperSignal West Dura Extended Duration Substrate as per manufacturer’s specifications (Pierce Chemical, IL). Images were captured using ChemiDoc XRS system (Bio-Rad, Hercules, CA).

### Insulin uptake by MCF-7 cells

MCF-7 cells transfected with either Sirt7 siRNA or mock were cultured overnight in chamber slides in MEM medium with a low (0.1%) FBS and at a confluency of 50%. Imaging of fluorescein isothiocyanate-insulin (FITC-insulin) uptake by MCF-7 cells transfected with either Sirt7 siRNA or mock were investigated using a fluorescence microscope (Leica TCS STED Confocal Microscope, Leica Microsystems Ltd., UK). Each experiment was carried out in triplicate.

## Results

**Figure 1:**
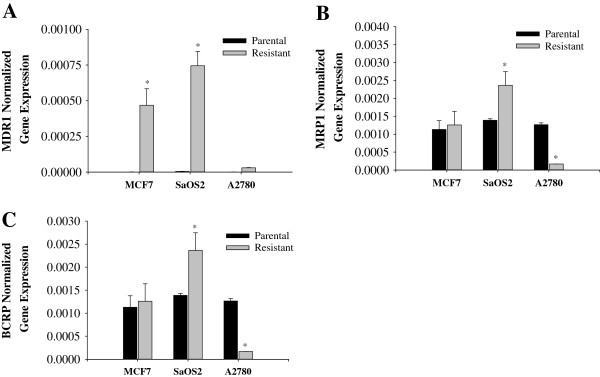
**mRNA expression of mdr1, MRP1 and BCRP.** mRNA expression measured by real time PCR of **A)** mdr1, **B)** MRP1 and **C)** BCRP in drug resistant cells and the respective parental cells normalized to GAPDH mRNA expression. mRNA expression of mdr1 is significantly higher in all Doxorubicin resistant cells (MCF-7 and SaOS2). No significant increase in mdr1 expression by Cisplatin resistant A2780 was observed.

**Figure 2:**
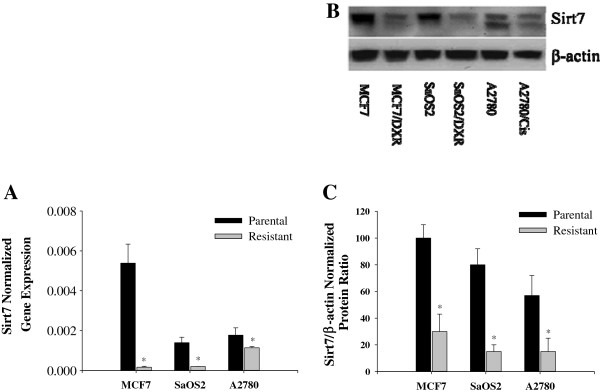
**mRNA and protein expression of Sirt7. A)** mRNA expression of Sirt7 normalized to GAPDH mRNA expression by RT-PCR, where the expression of Sirt7 is reduced in all chemoresistant cell lines tested (MCF-7, SaOS2, and A2780); **B)** A representative Western blot for Sirt7 protein expression showing a significant reduction of Sirt7 expression in all drug-resistant cells (MCF-7, SaOS2, and A2780); **C)** Densitometric quantitation of Sirt7/β-actin protein expression ratio.

The relationship between the expression of Sirt7 and P-glycoprotein, coded for by mdr1, BCRP, and MRP-1 was investigated using quantitative PCR in both the wild type cells and their respective drug resistant cell line. The mRNA expression levels of several drug resistance genes (mdr1: multi-drug resistance gene-1; BCRP: breast cancer resistance protein; and MRP1: multi drug resistance associated protein-1) in MCF-7/Dox, SaOS-2/Dox, and A2780/Cis were measured by real-time PCR. mRNA expression of mdr1 was consistently higher in the drug-resistant cells when compared to the parental cells (Figure 1A). The expression of MRP1 and BCRP was the same in MCF-7/Dox and parental cells, slightly upregulated in SaOS-2/Dox and inhibited in A2780/Cis (Figures 1A &1B). Sirt7, on the other hand, was inhibited in all the drug resistant cells examined (Figure 2).

**Figure 3:**
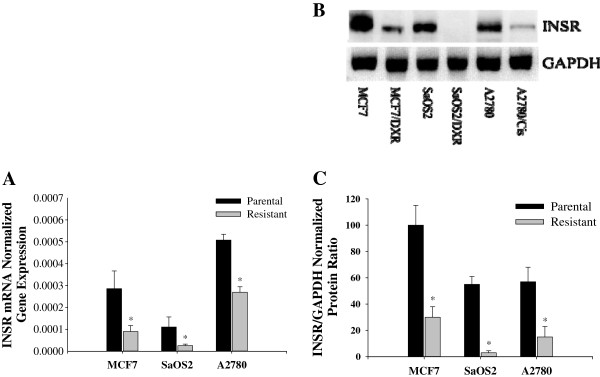
**mRNA and protein expression of INSR in drug-resistant cell lines. A)** mRNA expression of INSR normalized to GAPDH mRNA expression by RT-PCR; **B)** A representative Western blot for INSR protein expression showing a significant reduction of INSR expression in drug-resistant cell lines (MCF-7, SaOS2, and A2780); **C)** Densitometric quantitation of INSR/GAPDH protein expression ratio.

**Figure 4:**
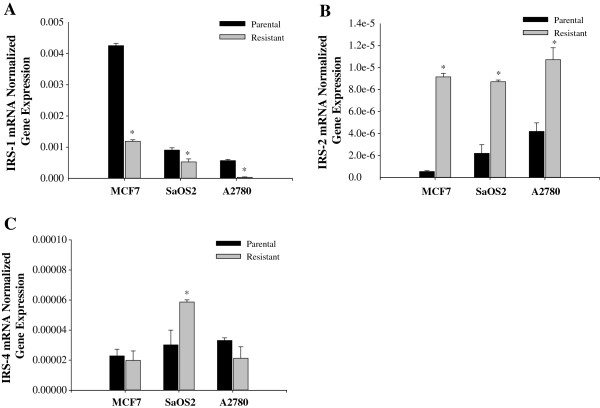
**mRNA expression levels of IRS-1, IRS-2 and IRS-4.** mRNA expression of **A)** IRS-1; **B)** IRS-2; and **C)** IRS-4 in drug resistant and their corresponding parental cells (MCF-7, SaOS2, and A2780).

**Figure 5:**
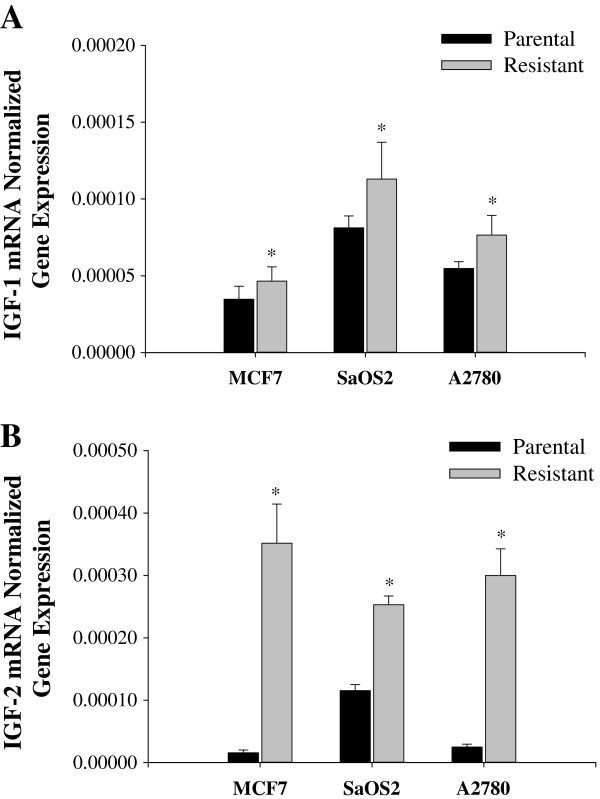
**mRNA expression levels of IGF-1 and IGF-2 in drug resistant and their corresponding parental cells.** mRNA expression of **A)** IGF-1 and **B)** IGF-2 in drug resistant and their corresponding parental cells (MCF-7, SaOS2, and A2780).

**Figure 6:**
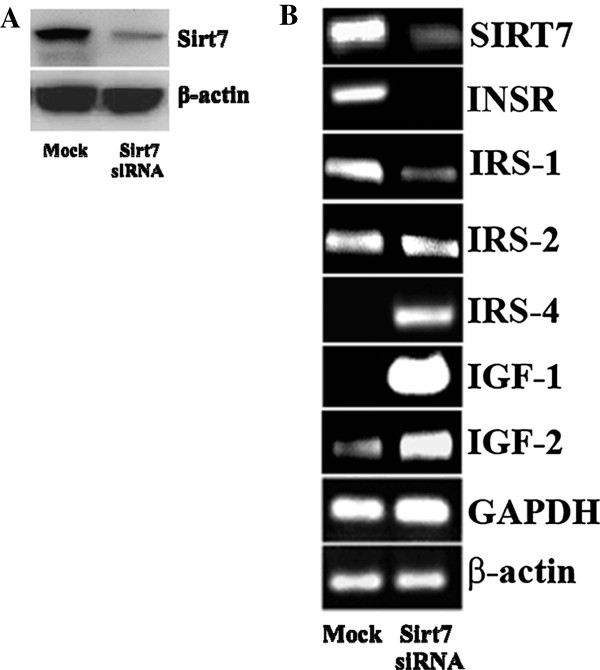
**Protein expression of Sirt7 and mRNA expression of insulin/IGF transduction pathway protein following inhibition of Sirt7. A)** Protein expression of Sirt7 following inhibition of Sirt7 in MCF-7 by siRNA; **B)** mRNA expression of Sirt7, INSR, IRS-1, IRS-2, IRS-4, IGF1, IGF2, GAPDH and β-actin in MCF-7 following inhibition of Sirt7 by siRNA.

**Figure 7:**
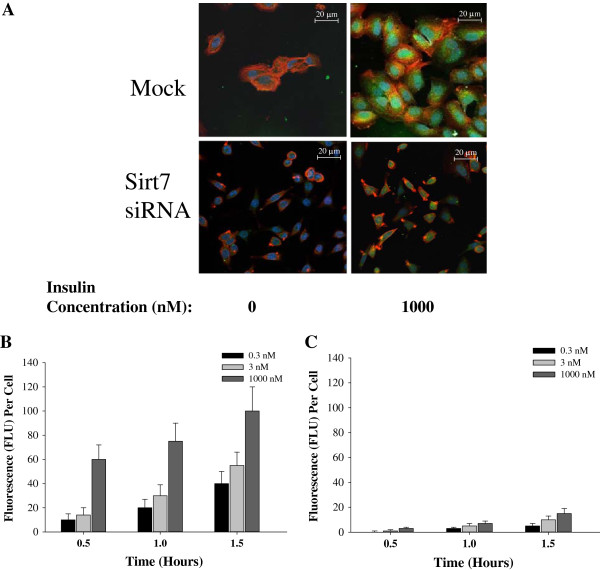
**FITC-conjugated Insulin uptake by MCF-7 following Sirt7 inhibition. A)** Representative image for FITC-conjugated Insulin uptake by MCF-7 transfected with either mock or Sirt7 siRNA vectors. Stably transfectcted MCF-7 cells were cultured in chamber slides in 0.1% FBS containing medium overnight. Cells were then incubated with FITC-conjugated Insulin (0 and 1000 nM) for 2 hours and then washed, fixed and analyzed by Leica confocal microscope. Nucleus was counterstained with DAPI (blue) while cytoplasm was counterstained with Rhodamine Phalloidin (red); **B)** Insulin uptake by MCF-7 Mock transfected and incubated with different FITC-labeled insulin for different time intervals; and **C)** Insulin uptake by MCF-7 cells transfected with Sirt7 siRNA.

To evaluate insulin/IGFs pathways in chemoresistance, we measured INSR, IRS-1, IRS-2, IRS-4, IGF-1 and IGF-2 mRNA. INSR was significantly inhibited in all the drug resistant cells when compared to their parental cell lines at the mRNA and protein levels (Figure 3). Although IRS-1 is a substrate most commonly observed in IGF-INSR binding with IGF-1 and IGF-2 [37], IRS-1 mRNA expression was inhibited in all the drug resistant cells lines (Figure 4A). However, IRS-2 mRNA expression was upregulated in all the drug resistant cell lines tested (Figure 4B). It is also known that IRS-4 is activated via IGF-1/IGF-INSR binding although the exact affect of IRS-4 is still unknown. As shown in Figure 4C, the mRNA expression of IRS-4 was not consistently affected in the drug-induced cell lines used in this study.The mRNA expression of IGF-1 and IGF-2 is consistently increased in all the cancer cell lines compared to their respective parental cells except MCF-7 cell line which did not show a significant increase in IGF-1 (Figure 5). This observation establishes an inverse correlation between the expression of IGF-1 and Sirt7, and a positive correlation between Sirt7 and INSR. To establish a causal relationship between Sirt7 and INSR inhibition and IGFs upregulation, Sirt7 in MCF-7 cells was inhibited with Sirt7 siRNA (Figure 6). Inhibition of Sirt7 significantly inhibited INSR and slightly upregulated IRS-1. It also upregulated the expression of IRS-4, IGF-1 and IGF-2. Insulin uptake was also inhibited following Sirt7 inhibition in MCF-7 (Figure 7). Sirt7 inhibition in MCF-7 resulted in inhibition of FITC conjugated insulin uptake when at added at lower concentrations (0.3-3.0 nM). However, a slight uptake was observed at 1 μM after 2 hours of addition. Insulin uptake by mock transfected MCF-7 cells was seen even at 0.3 nM (physiological) concentration with 30 min of incubation. This increased insulin uptake increased with time and concentrations. As expected, 10% FBS inhibited the uptake of insulin (data not shown).

## Discussion

Multi drug resistance (MDR) might be simultaneously involved in the participation of multiple genes and molecular pathways. Several mechanisms of MDR have been proposed, including the transporter-based MDR caused by the activation of transporter proteins such as P-glycoprotein (Pgp) and the non-transporter-based MDR, which is caused by altered activity of enzyme systems such as glutathione S-transferase π (GST-π). Expression of Pgp, GST-π and topoisomerase II were found to be useful for identifying drug resistance in gastric carcinoma [38]. Sirt7 expression was inversely correlated with the increased expression of the *mdr1* gene in drug-induced resistance. Sirt7 inhibition was also observed in Cisplatin-resistant cells, a non p-glycoprotein substrate, suggesting that the anti-apoptotic effect of Sirt7 inhibition is a non p-glycoprotein-dependent. Similarly, Sirt7 inhibition-induced drug resistance is a non-p53 mediated mechanism since the osteosarcoma SaOS-2 has *p53* gene rearrangements and deletions [39] and both have decreased Sirt7 expression. A recent study by which *Sir2* was overexpressed in drosophila flies resulted in promotion of caspase-dependent but p53-independent apoptosis [40].

Replicative senescence likely results from the shortening of telomeres to such an extent that the chromosome ends are not fully masked from recognition by the proteins responsible for double strand break repair. Once a critical shortened telomere length is attained, cell senescence is triggered. SIPS is not prevented by telomere elongation. It is accompanied by intense genetic instability with gross chromosomal abnormalities, possibly due to illegitimate DNA recombination, and is associated with relative inability to undergo apoptosis [41]. Recently, telomerase reverse transcriptase (*hTERT*) mRNA expression was shown to be significantly increased in gastric cancer, which was related with a worse differentiation and drug-resistance to Adriamycin [42]. Whether modulation of insulin/IGF pathways by Sirt7 inhibition could mediate hTERT expression is of interest and it is the subject of further investigation.

Our data demonstrate clearly that drug resistance is associated with upregulation of IGF/IRS-2 pathway through Sirt7 inhibition. Most metastatic cancers express higher levels of IRS-2 as compared to IRS-1. IGF-1 dependent activation of a cell causes enhanced IRS-2 signaling and increased IGF-I induced migration, adhesion and anchorage-independent growth. Moreover, decreased levels of IRS-2 significantly affected cells response to IGF-1 signaling. Although IRS-1 is the predominant substrate activated via IGF-1-mediated cell proliferation and protection from chemotherapy induced apoptosis, IRS-2 links between IGF-1 and integrins, leading to its metastatic behavior [43]. IGF-1 and IGF-1R activated cells have the ability to avoid apoptosis by regulating the actions of proteins such as caspase-3 and other apoptotic factors such as IL-3 and TNF-α [44,45]. Over expression of IGF-1R in *in vivo* and *in vitro* models of testicular and ovarian cancer inhibited Cisplatin-induced apoptosis [46]. In these tumors, however, induction of apoptosis by Cisplatin is not necessarily dependent on wild-type p53 [47-49]. Similarly, in human breast model (HBL100), IGF-1 protected cells against apoptosis induced by 5-fluorouracil, methotrexate, tamoxifen, or camptothecin [50].

Our study links Sirt7 to INSR and could explain the cytotoxic potentiating effects of insulin [51,52]. Insulin exerts this effect when it is given in combination with another chemotherapeutic agent. In MCF-7 human breast cancer cell line, methotrexate-induced cytotoxicity increased as much as 10,000-fold when combined with insulin [53]. Similarly, incubation of MDA-MB-231 with insulin resulted in an increased intracellular accumulation of the DNA-intercalating agent ellipticine and a concomitant increase in cytotoxicity. It was hypothesized that insulin imposes metabolic modification within cancerous cells, rendering them more sensitive to the effects of methotrexate while another study suggested an increase in the capacity to accumulate free intracellular methotrexate in MCF-7, a result of the increase in the intramembrane methotrexate transport system [54]. The cross-reaction of insulin with IGF receptors on cancer cell membranes increases the S-phase fraction in tumors, increasing the cells susceptibility to the cytotoxicity of anticancer drugs [6]. Addition of insulin to an asynchronous population of breast cancer cells increased the S-phase fraction to 66% compared to 37% in the controls [3]. Such an increase in the S-phase fraction would have a significant effect on the cytotoxicity of anticancer drugs, particularly the cell-cycle, phase-specific agents. Interestingly, Insulin differentiates selectively between cells of normal versus cancerous tissues. Insulin binds dominantly to tumor cells rather than to fat and fibrous tissue within tumors as demonstrated by autoradiographic studies [17]. Notably, breast cancer cell membranes have been found to have an average of seven times more INSR [55] and 10 times more IGF receptors [2] than normal breast and other tissues. Therfore, insulin predominantly targets cancer cells, with a relative sparing of host normal tissues. However, our data show clearly a significant inhibition of INSR expression by all the drug-induced resistant cells tested and as result would limit insulin potentiating therapy. Thus, drugs inducing Sirt7 may increase insulin cytotoxic potentiating effects by preventing INSR inhibition.

It is important to define the role of INSR in cellular response to stress with regards to development of drug resistance in cancer. Besides the possible discovery of a novel drug resistance mechanism, our results indicate that stress resistance in cancer shares common signaling pathways with that in aging. It would be of great interest to examine Sirt7/INSR pathway in aging. Since our data establish a causal relationship and implicate Sirt7 as a regulator of the insulin/IGF pathway, our data may also suggest a role of Sirt7 in hyperglycemia associated with chemoresistance.

## Competing interest

The authors declare that they have no competing interest.

## Authors’ contributions

All authors contributed to this work. AA and AS participated in the design of the study. AA, AS and SA conceived of the study, participated in its design. AA and AS performed the assays. AA drafted the manuscript and performed the statistical analysis. AS and SA revised the draft. All authors read and approved the final manuscript.
